# Realizing Clinical Trials with Astatine-211: The Chemistry Infrastructure

**DOI:** 10.1089/cbr.2019.3055

**Published:** 2020-08-13

**Authors:** Sture Lindegren, Per Albertsson, Tom Bäck, Holger Jensen, Stig Palm, Emma Aneheim

**Affiliations:** ^1^ Department of Radiation Physics and Targeted Alpha Therapy Group, Institute of Clinical Sciences, Sahlgrenska Academy, University of Gothenburg, Gothenburg, Sweden.; ^2^ Department of Oncology, Targeted Alpha Therapy Group, Institute of Clinical Sciences, Sahlgrenska Academy, University of Gothenburg, Gothenburg, Sweden.; ^3^ Department of Oncology, Region Västra Götaland, Sahlgrenska University Hospital, Gothenburg, Sweden.; ^4^ Cyclotron and PET unit KF-3982, Copenhagen University Hospital, Copenhagen, Denmark.

**Keywords:** astatine-211, targeted α, therapy

## Abstract

Despite the consensus around the clinical potential of the α-emitting radionuclide astatine-211 (^211^At), there are only a limited number of research facilities that work with this nuclide. There are three main reasons for this: (1) Scarce availability of the nuclide. Despite a relatively large number of globally existing cyclotrons capable of producing ^211^At, few cyclotron facilities produce the nuclide on a regular basis. (2) Lack of a chemical infrastructure, that is, isolation of ^211^At from irradiated targets and the subsequent synthesis of an astatinated product. At present, the research groups that work with ^211^At depend on custom systems for recovering ^211^At from the irradiated targets. Setting up and implementing such custom units require long lead times to provide a proper working system. (3) The chemistry of ^211^At. Compared with radiometals there are no well-established and generally accepted synthesis methods for forming sufficiently stable bonds between ^211^At and the tumor-specific vector to allow for systemic applications. Herein we present an overview of the infrastructure of producing ^211^At radiopharmaceuticals, from target to radiolabeled product including chemical strategies to overcome hurdles for advancement into clinical trials with ^211^At.

## Introduction

Targeted α therapy has for several decades attracted interest for therapy of disseminated cancer. The main rationale has been treatment of remaining minimal residual disease after primary treatments such as surgery, external radiotherapy, and/or chemotherapy.^1–4^ After the primary treatment, targeted α therapy has the potential to be a curative treatment for patients, given that proper care is taken when choosing the radioactive nuclide and treatment modality.

There are ∼400 (5-100%) α emitting radionuclides when including isotopes. However, among those only a few fulfill the criteria for nuclear medicine applications, that is, suitable half-life, absence of long-lived and/or toxic daughters, and feasible production of clinically relevant amounts. Narrowing the list to those with a half-life >1 h and no serial decay, which potentially will contribute to toxicity *in vivo*, only one nuclide remains, that is, astatine-211 (^211^At).

A few more radionuclides can be considered disregarding serial decay. These are thorium-227 (^227^Th), actinium-225 (^225^Ac), and radium-223 (^223^Ra).^5–7^ Including also nuclides with shorter half-lives, bismuth-213 (^213^Bi) and the lead-212/bismuth-212 (^212^Pb/^212^Bi) system can be added as potential choices.^8,9^ All these nuclides have been used in clinical settings but Ra[^223^Ra]-dichloride (Ra[^223^Ra]Cl_2_; Xofigo^®^) is the only clinically approved α-emitting radionuclide.^10^

However, when comparing the α-emitting radionuclides proposed for nuclear medicine applications in detail, ^211^At still emerges as one of the most promising nuclides. ^211^At can be produced relatively cost-effectively in reasonable yields with a medium energy cyclotron, comparable with the cost for cyclotron production of, for example, iodine-123 (^123^I). Despite the fairly straightforward production of ^211^At and a number of existing possible manufacturing sites, there are few facilities currently producing the nuclide. From this it can be concluded that the problem of ^211^At availability is not exclusively reflected by the possibility of radionuclide production. ^211^At needs to be converted into a chemically useful form after cyclotron production and it needs to be coupled to a carrier molecule. A chemical infrastructure must hence also be in place within a reasonable close proximity to the cyclotron production site.

To improve availability and motivate an increased production of the nuclide it is also essential to prove the potential of ^211^At-radiopharmaceuticals in clinical settings. So far there has been two minor clinical phase I trials completed with ^211^At-antibody radiopharmaceuticals, mainly financed by academic funding.^11,12^ The results from these studies indicate therapy potential with no or very mild toxicity related to the treatment. To further prove the clinical potential of ^211^At, including progress into phase II/III studies, substantial funding is required in which both academy and industry need to share the economic risk. However, normally the industry needs evidence of the clinical potential before taking financial risk. So, in addition to the described practical hurdles of ^211^At production and availability, the funding of such research additionally suffers from this chicken and egg dilemma.

It is important to note that the scarce use of ^211^At is not reflected by difficulties in any of the radiopharmaceutical production steps. The production, isolation, and chemistry are well documented and feasible. However, a complete radiopharmaceutical infrastructure is less widely spread.

In this study we will, despite the hurdles, show a working outline to produce ^211^At including a chemical infrastructure to provide activities needed for patient treatments, using existing chemistry.

## Astatine

Astatine, initially denominated “eka-iodine,” was first discovered in the United States in 1940 where it was produced at University of California, Berkeley through α-particle bombardment of natural bismuth.^13^ The astatine isotope with the longest half-life is astatine-210 (^210^At) with a half-life of 8.1 h. ^210^At, however, decays by polonium-210 (^210^Po, an α-particle emitter with a half-life of 138 d), making it unsuitable for medical purposes. Because of the lack of stable or long-lived isotopes, many chemical and physical properties of astatine are still not known but derived from its neighboring halogens.^14,15^

**FIG. 1.**
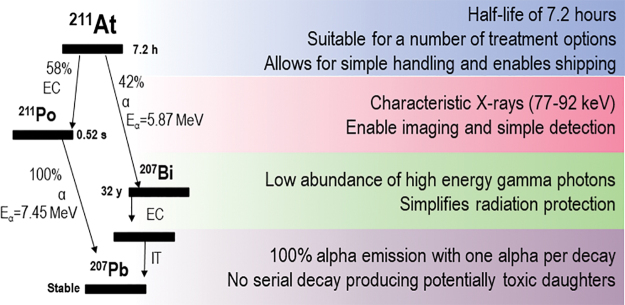
Physical properties of ^211^At. Color images are available online.

When comparing the physical properties of the α-emitting radionuclides proposed for nuclear medicine applications (^211^At, ^227^Th, ^225^Ac, ^223^Ra, ^213^Bi, ^212^Pb/^212^Bi) in detail, ^211^At emerges as perhaps the most promising alternative (Fig. 1). ^211^At decays with a half-life of 7.21 h in two branches: either by α-emission to bismuth-207 (^207^Bi) or by electron capture (EC) to polonium-211 (^211^Po). After the EC, ^211^Po promptly decays by α-emission to stable lead-207 (^207^Pb). This means that each decay will yield one α-particle. In addition, the EC branch to ^211^Po will yield characteristic polonium X-rays in the range of 70–90 keV. This enables simple quantification and the energy of the X-rays are in the range where it can be imaged with a γ camera.^16^

Astatine-209 (^209^At, half-life of 5.4 h) is another astatine isotope that decays in two branches either by α-emission to bismuth-205 (^205^Bi, 4%) or by positron emission (β^+^ decay) to polonium-209 (^209^Po, 96%). Due to high abundance of β^+^ it has been proposed as a theranostic pair to ^211^At.^17^ However, as ^209^At is produced by high-energy proton spallation of actinide targets,^18^ production of clinically relevant amounts can be foreseen to be problematic in a global perspective. In this sense, iodine-124 (^124^I) and ^123^I are more realistic theranostic pairs for ^211^At.

## Production and the Chemistry Infrastructure of ^211^At

**FIG. 2.**
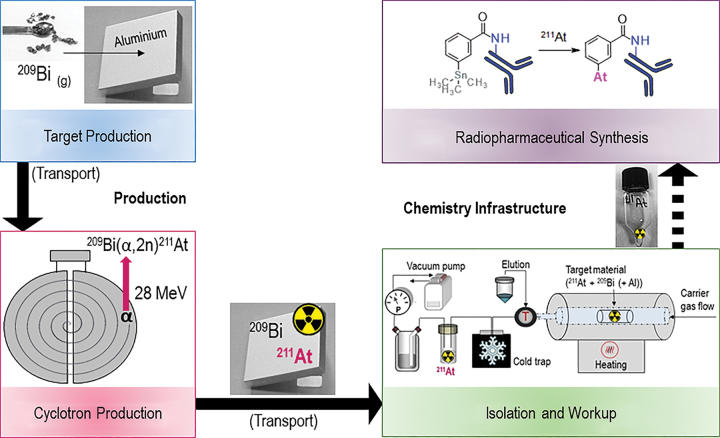
The chemical infrastructure for production of ^211^At radiopharmaceuticals. Color images are available online.

The overall infrastructure for producing ^211^At-radiopharmaceuticals can be divided into two main parts: (1) production, including targetry and cyclotron production of the nuclide followed by (2) the chemistry infrastructure, including isolation and work-up of ^211^At from the irradiated target and synthesis of the ^211^At-radiopharmaceutical. These steps are illustrated in Figure 2 and discussed in detail below.

### Targetry and cyclotron production of ^211^At

There are two possible routes for producing ^211^At, starting with a bismuth target: either indirectly through a ^211^Rn/^211^At generator in which ^211^Rn is produced by heavy ion irradiation of bismuth-209 (^209^Bi) through the nuclear reaction ^209^Bi(^7^Li,5n)^211^Rn,^18,19^ or directly through cyclotron production utilizing the nuclear reaction ^209^Bi(α,2n)^211^At.^20^ The advantage with the ^211^Rn/^211^At generator system is the longer half-life of ^211^Rn compared with ^211^At (14.6 h vs. 7.2 h). This will increase the distribution area of ^211^At from the production site with maintained astatine activity compared with the delivery of ^211^At alone. Although feasible, this route requires heavy ion high-energy beam irradiations and careful isolation and containment of the produced ^211^Rn, which is more technically demanding than the latter medium energy cyclotron production route. It is also possible to produce both ^211^At and ^211^Rn by high energy proton spallation of actinide targets but radiochemical yields from this production are to this date comparatively low.^21^

Although ^211^At can be produced by heavy ion irradiation or spallation, the main production route has been and still is through the ^209^Bi(α,2n)^211^At reaction. The reason for this is the relative simplicity of production, the comparatively high yields, and significantly higher number of available facilities. A previous review by Zalutsky and Pruszynski related to these issues and the worldwide potential of producing ^211^At showed that there are a number of existing medium energy cyclotrons around the world with the capacity to produce ^211^At.^22^ However, many of the cyclotrons mentioned in that review are old and some of them have been or are under decommissioning. In addition, a few new medium energy cyclotrons have recently been installed or are under installation and cyclotron models that can produce ^211^At are today available for purchase “off the shelf” from, for example, IBA, Belgium and Sumitomo, Japan. Because of this, an updated, nonexhaustive, worldwide list of cyclotrons capable of producing astatine is provided in Table 1. Astatine production on a regular basis is today mainly pursued in Copenhagen, Denmark, Nantes, France, several sites in Japan (Fukushima, Osaka, Takasaki, Chiba, and Wako Saitama) and at two facilities in the United States, Seattle, Washington, and Durham, North Carolina.

**Table 1.** Facilities with Cyclotrons Capable of Producing ^211^At

Location	Institute	Cyclotron model
Europe		
Copenhagen, Denmark	Copenhagen University Hospital	MC-32 Scanditronix
Oslo, Norway	University of Oslo	MC-35 Scanditronix
Nantes, France	Arronax	Cyclone 70, IBA
Orleans, France	CNRS—CEMHTI laboratory	THOMSON-CSF
Rez, Czech Republic	Czech Academy of Sciences	U-120M
Dubna, Russia	JINR—FLNR	U200
Tomsk, Russia	Tomsk polytechnic University	U-120
Warsaw, Poland	Heavy Ion Laboratory University of Warsaw	U200-P (new installation planned)
Cracow, Poland	IFJ-PAN Cyclotron Centre Bronowice	AIC-144
Groningen, The Netherlands	University of Groeningen	AGOR cyclotron
Birmingham, United Kingdom	University of Birmingham	MC-40 Scanditronix
Jyväskylä, Finland	University of Jyväskylä	AVF K130
Brussels, Belgium	VUB	CGR-MeV model 560
Jülich, Germany	Forschungszentrum Jülich	Cyclone 30 XP, IBA
North America		
Durham, United States	Duke University	The Cyclotron Corporation CS-30
Seattle, United States	University of Washington	Scanditronix MP-50
Philadelphia, United States	Penn Medicine	Japan Steel Works (JSW) BC3015
Davis, United States	Crocker Nuclear Laboratory, UC Davis	In-house 76-inch isochronous cyclotron
College Station, United States	Texas A&M	K500 Superconducting Cyclotron
Bethesda, United States	NIH	The Cyclotron Corporation CS-30
Ann Arbor, United States	University of Michigan	The Cyclotron Corporation CS-30
Asia		
Daejeon, South Korea	IBS-RAON	New installation planned—Cyclone 70, IBA
Osaka, Japan	RCNP-Osaka University	K140 AVF/K400 ring cyclotron
Takasaki, Japan	QST-Takasaki, (TIARA)	AVF (K110)
Chiba, Japan	QST—NIRST	AVF-930
Fukushima City, Japan	Fukushima Medical University	CYPRIS MP-30
Wako Saitama, Japan	RIKEN—Nishina Center for Accelerator-Based Science	AVF
Sichuan, China	NSE-SCU, Sichuan University	The Cyclotron Corporation CS-30
Africa		
South Africa	iThemba Labs	SPC1/SPC2

The excitation function for the ^209^Bi(α,2n)^211^At reaction shows a maximum production yield using an α-beam of 31–35 MeV of the incident α particle. However, the energy should be kept at ∼28 MeV to avoid or minimize the competing reaction ^209^Bi(α,3n)^210^At. Coproduction of the ^210^At is unwanted because of its decay to the potentially toxic daughter ^210^Po.^23^

There are several advantages by using natural bismuth as a target for producing ^211^At. As bismuth is readily abundant, there will not be any lack of target material, which enables a theoretical indefinite production of ^211^At. This can be compared with several other α-particle emitters such as ^225^Ac, ^213^Bi, and ^223^Ra, where nuclear legacy material has been initially used for production and where future targetry and/or production of clinical amounts is either limited in availability or complicated by the handling of the starting material. ^24–27^

There are two main target set-ups for producing ^211^At from bismuth, either thin targets for internal cyclotron irradiations or thick targets for extracted beam irradiations.^20,28^ For thick target preparation the bismuth can, for example, be electroplated or melted on the backing^29^ or used as granulates that are melted during irradiation.^30^ Thin targets are mainly prepared using physical vapor deposition of the bismuth on a backing material.^31^ In any case, targetry for the production of ^211^At is a relatively straightforward and cost-effective process. However, one difficulty encountered with using bismuth as target material is its fairly low melting point (271.4°C). This in combination with a low melting and boiling point (Bp) of astatine (302°C and 337°C, respectively) means that an effective cooling of the targets and measures to enclose potentially evaporated astatine during the cyclotron irradiation are required. Therefore, the targets often have liquid cooling on the backside and front-side gas cooling.^32^ In addition, melting of bismuth and evaporation of ^211^At during thin target irradiation can be prevented by application of a fine layer of aluminum on top of the bismuth layer.^33^

The efficacy in cyclotron production of ^211^At is related to the helium ion source, the α-beam energy, the current of the beam, and the cooling of the target. These parameters need to be optimized, taking into account the described physical properties of bismuth and astatine.

As for today the most efficient and highest yields of ^211^At production reported come from Zalutsky et al. in Durham, NC.^32^ They reported a maximum produced amount of ^211^At of 6.6 GBq at end of bombardment. Providing an efficient chemistry this would allow clinical radiopharmaceutical production for several patients in a single production run, in addition to transport within an area corresponding to several hours of decay from the production site.

### Isolation and work-up of ^211^At from irradiated targets

After production ^211^At embedded in the bismuth layer of the target must be isolated and converted into a chemically useful form for further chemical processing, that is, labeling and production of astatinated molecules. The isolation from the activated target can be performed either by wet extraction or by dry distillation.^33,34^ Using the wet extraction method, the irradiated target is partially or fully dissolved in strong acid, for example, nitric acid.^35^ The ^211^At is then recovered using solvent extraction into an immiscible organic phase.^36^ Before the solvent extraction step, it can be necessary to change the aqueous phase by distillation of the dissolution acid. In general the astatine also has to be back extracted from the organic phase or the solvent changed to achieve a chemically useful form for further labeling.^29^ This sequential manual processing results in good yields but is time-consuming compared with the short half-life of the nuclide.^29,37^ An alternative method to solvent extraction after target dissolution is astatine adsorption on a solid support followed by elution.^37^

In dry distillation the irradiated target is heated well above the Bp of ^211^At (Bp = 337°C) releasing it from the bismuth (Bp = 1564°C), which remain as a solid/liquid. The evaporated ^211^At can then be condensed in a cold trap or captured through gas scrubbing utilizing different solvents.^38^ Before the distillation it is advantageous to remove the activated bismuth layer from the aluminum backing of, for example, a thin target, as this allows the use of a small distillation unit that can fit in a hot cell or a glove box. Furthermore, reducing the volume of target material and preheating the distillation furnace will speed up the subsequent dry distillation, that is, the evaporation of ^211^At. In this way, dry distillation can be completed within 2 min.^33^ Furthermore, if capturing the ^211^At in a cold trap (as a dry residue) it can be eluted in a small volume of a preferred solvent. In this way the overall preparation time, distillation, and work-up, can be limited to <20 min from the start of the distillation.^33^

After distillation care should be taken when selecting the eluting solvent and to the following activity concentration. Chloroform has been found to be a suitable solvent for ^211^At. It readily solvates high activities of the nuclide and the resulting activity in the solvent is stable for at least a couple of hours. The high vapor pressure of chloroform enables evaporation of the solvent without loss of ^211^At activity, leaving the astatine as a dry residue. Although the ^211^At speciation in chloroform is not known, recent results suggest that radiolysis products of chloroform such as chlorine species may be important for the apparent stability of ^211^At in this solvent.^39^ However, it should be noted that distribution ratios for ^211^At into chloroform from nitric acid are low, making this solvent unsuitable for astatine recovery using wet extraction.^15^

Another option for eluting ^211^At from the cold trap is to use sodium hydroxide or an aqueous solvent containing a reducing agent to define an astatide state.^14^ However, by introducing a reducing agent the subsequent halogen chemistry might be compromised. Yet another option is to use methanol. Contrary to chloroform, this may allow for direct labeling of protein conjugates but activity eluted in this solvent should be used immediately as ^211^At is not fully stable in methanol. Introducing an oxidizing agent, for example, a halogen-based oxidizing agent such as *N*-chlorosuccinimide, has been found to increase the stability of ^211^At in methanol.^40,41^

In comparison, the dry-distillation method shows some advantages over manual wet-extraction methods. Besides generally faster isolation of ^211^At and hence also higher yields, distillation configurations with a cold trap enable elution of the ^211^At in small volumes ready for immediate use in the subsequent chemistry.^33^

Independent of method, the isolation and work-up procedure should be performed as to minimize risk of internal contamination from the potentially volatile α-particle emitter, that is, in a sealed environment such as a negative pressure glove box or hot cell. Radiation protection for personnel from the decay of ^211^At in terms of external shielding, lead-glassed glove box window, or similar, is less important because of the low abundance of high-energy gammas [^211^At (687 keV) and ^211^Po (569.7 and 897.8 keV)] besides ^211^Po X-rays (70–90 keV), giving low external dose.

### ^211^At radiopharmaceutical synthesis

This section provides an overview of the different chemical approaches to produce astatinated radiopharmaceuticals. Much of the chemical development of ^211^At compounds is based on previous inorganic and organic chemistry of astatine and for more detailed information on this topic we like to refer to a comprehensive review by Guerard et al.^42^

Astatine is the second heaviest element of the halogen group and it possesses many similar characteristics compared with its neighbor, iodine. In general, halogen properties can be used in astatine-labeling chemistry but there are also some obvious differences between astatine and iodine. For example, unlike iodine, astatine cannot be stably coupled to tyrosine residues of proteins. Instead of binding to tyrosine ^211^At has been found to form weak bonds with the sulfhydryl groups of cysteine.^43^ This apparent instability of the direct ^211^At-protein bond compared with iodine can be ascribed to the fact that astatine, besides its halogen character, also displays some metallic properties.^44^ The metalloid behavior of astatine has led to a few investigations in which its chelating possibilities were evaluated.^45,46^ Although demonstrating the metallic properties, no chelate has so far resulted in a sufficiently stable bond with astatine. The development of radiopharmaceuticals with astatine has therefore, instead of using a metal-like chelate, mainly been directed to the formation of covalent bonds such as the aryl–astatine bond. Several routes, based on iodine chemistry, for synthesizing aromatic astatine compounds, have been investigated. These chemical reactions include halogen exchange,^47^ diazonium salt reactions,^48^ aryliodonium salt reactions,^49^ and electrophilic substitution of metal-functionalized aromatic compounds.^50,51^ Recently reactions involving boronic esters and boronic acids as leaving groups for aromatic astatination of biomolecules have also been explored.^52^ Among the functionalized aromatic compounds, the most common strategy so far has been aryl–tin agents in which the ^211^At is introduced as an astatine–carbon bond in the aryl ring through an electrophilic destannylation reaction.^51,53,54^

It should be noted that the bond strength of the astatine–carbon bond has been calculated to be weaker than the corresponding iodine–carbon bond.^55^ The stability of the ^211^At-aryl carbon bond was recently evaluated by Teze et al. and the results showed that the apparent instability can be attributed to oxidative decomposition, which *in vivo* particularly occurs in lysosomes after cell internalization.^56^ This also relates to the nonresidualizing properties of halogens and may explain the low *in vivo* stability of small ^211^At-labeled molecules, which after internalization processes in the kidney are decomposed, resulting in systemic reabsorption of free ^211^At.^57^ To overcome the hurdle of exocytosis of ^211^At after internalization, a guanidinomethyl-containing reagent that increases intracellular retention of halogens (i.e., iodine and astatine) was developed by Zalutsky and colleagues.^58^ The guanidine residue of the astatinated version of this reagent is postulated to sterically prevent cleavage of the ^211^At carbon bond after cell internalization.^57,59^ Pyridine instead of aryl derivatives have also been used in protein halogenation in attempts to increase intracellular retention of ^125^I and ^211^At.^60,61^

**FIG. 3.**
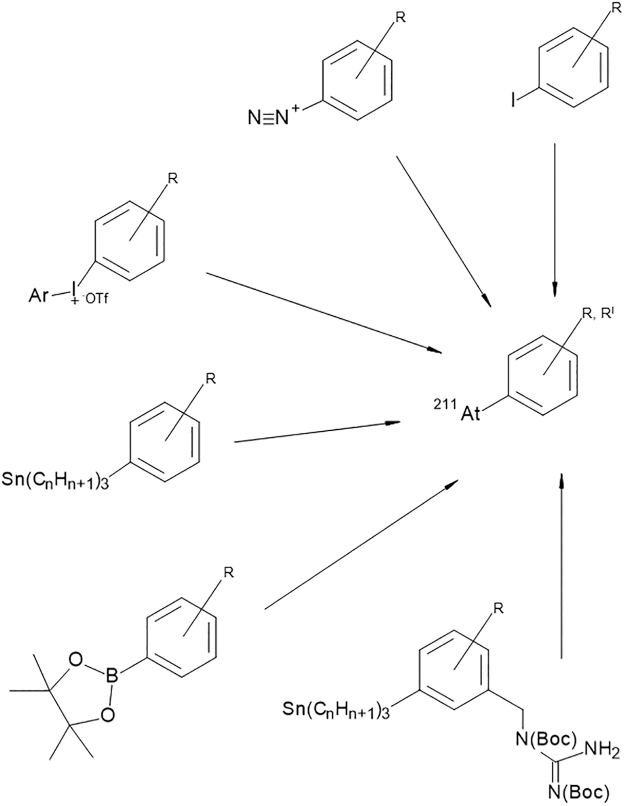
Examples of different aryl functional groups for ^211^At substitution reactions. From top: Iodine, isotope exchange; diazonium salt; aryliodonium salt; aryl tin; aryl boron pinnacol ester; aryl tin with guanidine moiety.

An overview of the different aromatic labeling strategies is given in Figure 3.

Despite the relative instability there are some recent reports on aromatic ^211^At compounds showing favorable *in vivo* distribution, for example, [^211^At]-meta astato benzylguanidine,^62^ ^211^At-labeled poly (ADP-ribose) polymerase (PARP) inhibitor,^63^ and ^211^At-labeled sigma receptor ligands.^64,65^ IgG antibodies have been the main vector for targeted α therapy using ^211^At and although labeled by aromatic reagents, the ^211^At antibodies show relative stability *in vivo*, particularly for intracompartmental applications.^11,12^

Higher bond energies compared with the ^211^At-carbon bond have been shown between boron and astatine.^66^ Based on this chemistry, Wilbur et al. developed and investigated several boron cage reagents, nido- and closo-carboranes, for radiohalogenation of biomolecules.^67,68^ These reagents have also been implemented in protein labeling and were found to be more stable *in vivo* compared with the corresponding astatinated proteins labeled by aromatic reagents.^67,69,70^

**FIG. 4.**
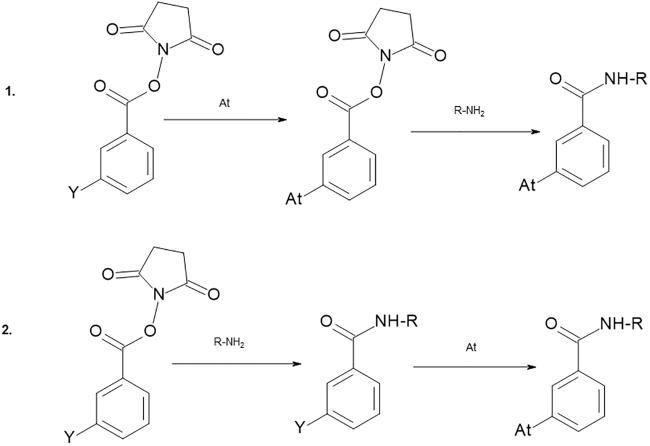
Methods for astatine labeling through a bifunctional reagent carrying an *N*-succinimidyl ester group for conjugation and a reactive group Y for astatination. **(1)** Two-step radiochemical reaction, labeling of the reagent followed by astatination. **(2)** One-step radiochemical reaction, conjugation of the reagent followed by astatination of the conjugate.

As previously mentioned, ^211^At cannot be labeled to tyrosine residues of proteins and an intermediate bifunctional reagent utilizing one of the above-detailed astatination strategies is therefore required. It commonly includes an *N*-succinimidyl ester, isothiocyanate, or a maleimide moiety, which can be coupled to amines or sulfhydryl groups of proteins and peptides.^54,71–73^ The conjugation of these bifunctional reagents to proteins can, depending on the nature of the reagent, be performed in two routes: either two radiochemical steps, astatine labeling to the bifunctional reagent followed by conjugation of the astatinated reagent to the protein, or one radiochemical step where preconjugation to the protein is performed before the astatine reaction (Fig. 4). By preconjugation the protein has been made susceptible for astatination, which is similar to that of labeling with metallic radionuclides where chelates are preconjugated to the protein. When applicable, the later route presents many advantages over the two-step approach, for example, higher radiochemical yields and higher specific activities can routinely be produced.^54,67^ In addition, the radiochemical reaction can be completed within a few minutes consequently reducing the absorbed dose in the reaction volume. Including purification, the ^211^At radiopharmaceutical product can be prepared within 20 min. Using this type of labeling method in combination with recovery from dry distillation, the overall radiochemistry can be completed in <1 h.

### Automatic radiopharmaceutical production

The scarce availability of ^211^At is not only related to available cyclotrons and cyclotron production capacity. It is also related to the chemistry infrastructure including isolation/work-up after cyclotron production and the subsequent synthesis, which, because of its half-life, needs to be in place within a reasonably short distance to the cyclotron. At the moment all ^211^At research is based on custom systems for recovery after cyclotron production followed by manual synthesis methods. To meet the anticipated future demand for clinical trials and to increase basic and preclinical research with ^211^At, the chemistry infrastructure as a whole needs to be developed and significantly improved beyond these systems. One such improvement would be automatic pharmaceutical systems adapted to ^211^At research, that is, automatic systems for isolation and work-up of ^211^At after cyclotron irradiation followed by automatic synthesis of ^211^At radiopharmaceuticals. Automation would not only facilitate the synthesis of ^211^At compounds and radiopharmaceuticals but also increase the safety of handling ^211^At and increase reproducibility in pharmaceutical production by reducing the necessity of hands-on manual work and risk of human error.

A challenge for general automation lies in the difference in target configuration between different astatine-producing cyclotrons. The target configuration affects the design of the systems for recovery of ^211^At from irradiated bismuth targets and is one reason for the existence of different types of custom systems. One way to work around this problem would be to customize an automatic system for a specific target/cyclotron, providing access to astatine and astatinated compounds for several research groups centered around one cyclotron. Another way of circumventing the problem with differences in target configurations is to remove the activated bismuth layer from the backing. This provides a relatively similar starting point for automation independent of the original target design. As mentioned earlier, such a solution also allows for the use of a compact heating system in the recovery of ^211^At using dry distillation, meaning that the entire automatic system including recovery, work-up, and synthesis, more easily can fit into a glove box or hot cell.

There are three reports on automatic systems for astatine recovery: one platform for the complete synthesis from irradiated target to labeling of biomolecules using dry distillation,^74,75^ and two automatic systems for converting ^211^At into a chemically useful form by wet extraction.^37,76^ However, at present, there is no commercial automatic system available for isolation of ^211^At after cyclotron production and the production of ^211^At radiopharmaceuticals.

The automatic dry-distillation system utilizes a tube furnace and quartz glass setup for astatine evaporation where volatilized ^211^At is confined and moved within the system using a reduced pressure applied on the outlet of the system and a light flow of nitrogen on the inlet. The target material is removed from the backing before distillation and is inserted into a preheated distillation furnace. In this way the evaporation process is completed in <30 s. The evaporated ^211^At is condensed in a cold trap containing a system of capillary tubing and a valve placed between the furnace and cold trap to allow for elution of the activity throughout the capillary. After distillation the recovery of ^211^At from the activated bismuth is routinely 80%–90%. After recovery from the target the isolated ^211^At then enters the conjunct synthesis module where steps for synthesis are included such as reagent introduction and purification, which to a high extent depends on the radiopharmaceutical to be produced. The complete setup is controlled by preprogrammed software that can be combined with input from online activity monitoring of the ^211^Po X-rays from ^211^At decay.^75^

An automatic wet extraction system for recovery of ^211^At from irradiated bismuth targets was recently reported by O'Hara (US 2018/0308599 A1).^76^ In this system, the target is mounted in a target dissolution block in which parts of the target and produced ^211^At are dissolved by nitric acid. The nitric acid is then distilled and the activity redissolved in hydrochloric acid before being and loaded onto a resin column. The column is washed with an acidic wash solution followed by elution with an eluting solvent. The complete procedure from dissolution is controlled by software. Higher radiochemical yields of ^211^At isolated from the target and an overall simpler procedure is achieved with this method compared with previously reported manual wet extraction methods.

A similar automated wet extraction method was evaluated by Li et al.^37^ The difference from the method reported by O'Hara is mainly the solid support, which in this case is based on tellurium. The irradiated target is dissolved by nitric acid and the nitric acid is converted to hydrochloric acid by addition of hydroxylamine hydrochloride. The solution is then loaded onto the tellurium column. The column is washed and the ^211^At is eluted with sodium hydroxide. High yields were obtained within a 90- to 100-min preparation time.

The advantage with this system is that it is somewhat less reliant on the target configurations of the cyclotrons compared with the described dry-distillation method.

## Clinical Experience with ^211^At

At Fred Hutchinson Cancer Research Center (Seattle, WA), there is currently a clinical phase I/II study enrolling patients with acute myeloid leukemia and acute lymphoblastic leukemia for treatment with an anti-CD45 antibody, ^211^At-BC8-B10, before donor stem cell transplant. The study aims to determine side-effects and the best dose of ^211^At-BC8-B10. There are no results reported from this study yet (NCT03128034).

There are two completed phase I clinical studies using ^211^At-labeled antibodies: one as a postsurgery boost treatment for recurrent brain tumor and another with intraperitoneal treatment of ovarian cancer patients having relapsed after primary treatment.^11,12^ Both these intracavitary studies showed mostly mild (grade I–II) side-effects across all dose levels from the treatment. In both studies, the antibodies were labeled using aromatic bifunctional agents with Stannyl-based according to Figure 3.

In the brain cancer study, performed at Duke University, Durham, North CA, 71–347 MBq ^211^At-labeled chimeric anti-tenascin monoclonal antibody 81C6 was given locally in the resection cavity followed by salvage chemotherapy. Results from 18 treated patients showed no dose-limiting toxicity. A few patients experienced low-grade neurotoxicity but no grade 3 toxicity was related to the treatment. The study showed that such a local intracavitary therapy in this dose range is safe and may result in a prolonged survival for the patients.^11^

In the ovarian cancer trial performed in Gothenburg, Sweden, patients were treated intraperitoneally with ^211^At-MX35(Fab)_2_ antibody fragment, targeting the sodium-dependent phosphate transport protein 2B (NaPi2b). Nine women who had relapsed after standard primary treatment were, after treatment by salvage chemotherapy in complete, or near complete, remission, subsequently treated with ^211^At-MX35(Fab)_2_ in 1–2 L of peritoneal dialysis fluid (icodextrin 7.5%). By blocking the sodium/iodide transporter with potassium perchlorate in sodium iodide symporter-expressing organs such as the thyroid and stomach, a significant reduction in uptake of free ^211^At in the thyroid could be recorded. After the initial nine patients, an additional three patients were treated after a change in the protocol regarding preparation of the radiopharmaceutical. The synthesis route was simplified through preconjugation of the antibody fragments compared with previous two-step labeling, as given in Figure 4, enabling higher radiochemical yields and consequently higher activity preparations. The results from the 12 patients in total showed that there was little escape of activity from the peritoneal cavity during a 48-h time period, that is, most of the ^211^At activity decayed in the abdominal cavity. Dosimetric evaluation of organs at risk showed that this treatment can be delivered without causing any acute toxicities. Indeed, most toxicities recorded were low grade (I–II) and mainly related to the treatment procedure, with no observed hematological toxicity, thus no dose-limiting toxicity was reached.^77^

Despite low risk of acute toxicity from ^211^At therapy, long-term stochastic risks may arise and should be considered, particularly for an upfront adjuvant therapy.^78^ Weighing dose to various size microtumors^79^ with recorded patient dosimetry^12^ and calculated long-term stochastic risks,^78^ an activity concentration of 200 MBq/L of ^211^At-labeled antibody may be a justifiable dose in future clinical intraperitoneal trials.^77^

## Discussion

Targeted α therapy is an emerging strategy for treatment of cancer, where the main rationale is treatment of small-scale disease, considering the high linear energy transfer and short path length of the α-particles. Therefore, α-particle therapy can be envisioned as an adjuvant treatment after standard primary treatments with, for example, surgery and chemotherapy. However, no such upfront targeted α-therapy setting for minimal residual cancer exists today. The only approved α-emitting radiopharmaceutical today is Xofigo, that is, [^223^Ra]RaCl_2_, which is used for palliative treatment of bone pain resulting from skeletal metastases in disseminated castrate-resistant prostate cancer.^80^ Another α-emitting radionuclide that currently is intensively investigated for treatment of cancer is ^225^Ac.^81,82^ Similar to ^223^Ra, ^225^Ac has frequently been clinically used on late-stage castrate-resistant prostate cancer.^83,84^ Yet another radionuclide that has recently attracted interest for targeted α-therapy is ^227^Th, the rationale being an *in situ* generator of ^223^Ra.^85^ At present, patients are enrolled in a phase I study for treatment of certain mesothelin-expressing cancers using ^227^Th (BAY2287411).

These radionuclides, ^227^Th, ^225^Ac, and ^223^Ra, are serially decaying radionuclides and moving treatment upfront to treat minimal residual metastases into a curable therapy would not be feasible. It is difficult to control the radioactive daughter distribution after decay of these radionuclides and therefore it may result in increased normal tissue toxicity and an increase in the risk for secondary cancer development.^86–88^

At present, ^211^At is the only radionuclide for targeted α therapy that can foreseeably safely meet the rationale of upfront treatment on minimal residual cancer. “Upfront” here means treating patients in complete remission after, for example, surgery and chemotherapy, before any sign of relapse. If the relapse frequency is <100%, this suggests that already-cured patients would be subjected to the targeted α therapy. ^211^At only releases one α particle per decay and does not result in any toxic α- or β^−^-emitting daughter nuclides. From toxicological and dosimetric perspectives, this is advantageous over the serially decaying nuclides described previously. However, one α-particle per decay means that less energy per nuclide can be deposited at or near the tumor site, compared with multiple α-particle-decaying radionuclides, and suggests that higher administered activities would be required.

^211^At has been applied in two clinical studies.^11,12^ In Gothenburg, Sweden, 12 ovarian cancer patients were treated intraperitoneally with ^211^At-MX35(Fab)_2_ in a phase I study. Results indicate a well-tolerated treatment with no side-effects from the radiation.^77^ The results proposed that calculated curative doses would be safe and adequate to administer to patients using existing labeling chemistry.^79^ Based on the results from the phase I study, initiation of an upfront phase II/III trial to determine the effect of the treatment is motivated.

A phase II/III trial including a large number of patients will require improvement in the availability of ^211^At and a highly developed chemical infrastructure. This means that besides targetry and cyclotron production, an efficient system for converting ^211^At into a chemically useful form and subsequent chemical synthesis to produce the radiopharmaceutical are required.

A common way of facilitating chemical synthesis in nuclear medicine applications is automation utilizing radiopharmaceutical synthesizers, for example, in the production of [^18^F] FDG or ^68^Ga-DOTA-octreotate. In this way the synthesis will facilitate reproducible and repeatable production. An automatic approach would also be a possible solution to the chemistry infrastructure problem presently limiting ^211^At research. A system that automatically converts ^211^At into a chemically useful form after cyclotron production^37,75^ and automatically produces the radiopharmaceutical^74^ would meet the safety requirements in handling ^211^At and facilitating larger clinical trials and multicenter studies. Standardization and reliable quality production of ^211^At and a chemical infrastructure is a requirement for future routine hospital production of ^211^At radiopharmaceuticals. One may envision several different approaches for implementing the logistics of the supply of ^211^At and the subsequent synthesis of the ^211^At radiopharmaceutical. Besides optimization and improvement of the cyclotron production, a delivery chain must also be established. Target distribution from cyclotrons to surrounding treatment centers within a circa one half-life radius followed by automatic on-site production would most likely allow for the best use of the produced activity, limiting radiolysis of the product. In densely populated areas, or where larger hospital and producing cyclotrons are located in close proximity, target work-up and pharmaceutical synthesis at the cyclotron facility may also be an alternative.

An additional issue that needs to be addressed is the apparent *in vivo* instability of ^211^At compounds, particularly important for general systemic applications with this nuclide. Although relatively high stability of astatinated compounds is often observed *in vitro*, deastatination may occur *in vivo*, predominately when small targeting vectors that are metabolized by the kidneys are used.^56,89^ The reabsorption of free ^211^At after sequestering of ^211^At molecules by the kidneys is believed to be related to intracellular oxidative decomposition after exocytosis and subsequent systemic recycling.^56,90^

There are intense efforts made to improve the *in vivo* stability of ^211^At compounds to enable general nuclear medicine applications.^66,91,92^ Many of the hampering features of ^211^At relate to its halogen characteristics. Like iodine, astatine is nonresidualizing after cell internalization and instability is to some extent related to intracellular decomposition and exocytosis of free ^211^At. Efforts to improve retention of ^211^At intracellularly have been made by utilizing different modifications of the aryl ring.^58,61^ Despite the stability issue, relative stability of a few ^211^At compounds, proteins, or small molecules labeled by aromatic intermediates have been observed and may be explained by fast kidney clearance in relation to slow or noninternalization properties.^63–65,93^ In addition, ^211^At-labeled antibodies synthesized with existing well-established chemical methods show sufficient stability in intracavitary applications and allow for treatment in several different cavity modalities such as intraperitoneal, intrathecal, and in the blood compartment following, for example, leukemia or lymphoma.

## Conclusion

In this review we show that despite existing challenges, there have been major steps taken in research for nuclear medicine applications with ^211^At, that is, the feasibility of producing ^211^At and the implementation of systems for target work-up and chemical synthesis. By providing a worldwide supply chain of ^211^At utilizing existing and new installations of cyclotrons and implementation of an efficient chemistry infrastructure, the main obstacles with this nuclide can be overcome and hence the demand concerning ^211^At radiopharmaceuticals for research and clinical trials could both be met and significantly enhanced.
